# Recent changes in the Dutch foodscape: socioeconomic and urban-rural differences

**DOI:** 10.1186/s12966-020-00944-5

**Published:** 2020-03-20

**Authors:** Maria Gabriela M. Pinho, Joreintje D. Mackenbach, Nicole R. den Braver, Joline J. W. Beulens, Johannes Brug, Jeroen Lakerveld

**Affiliations:** 1 Department of Epidemiology and Biostatistics, Amsterdam Public Health Research Institute, Amsterdam UMC, Vrije Universiteit Amsterdam, de Boelelaan 1089A, 1081 BT Amsterdam, The Netherlands; 2 Julius Centre for Health Sciences and Primary Care, University Medical Centre Utrecht, Universiteitsweg 100, 3584 CG Utrecht, The Netherlands; 3 Amsterdam School of Communication Research (ASCoR), University of Amsterdam, P.O. Box 1, 3720 BA Bilthoven, the Netherlands; 4 Faculty of Geosciences, Utrecht University, Princetonlaan 8a, 3584 CB Utrecht, The Netherlands

## Abstract

**Background:**

Obesogenic food environments may influence dietary behaviours and contribute to obesity. Few countries quantified changes in their foodscape. We explored how the availability of different types of food retailers has changed in the Netherlands across levels of neighbourhood socioeconomic status (SES) and urbanisation.

**Methods:**

This longitudinal ecological study conducted in the Netherlands had as unit of analysis administrative neighbourhoods. From 2004 to 2018, the geographic location and type of each food retailer were objectively assessed by a commercial company. Food retailers were categorised as local food shops, fast food restaurants, food delivery, restaurants, supermarkets, and convenience stores. Information on neighbourhood SES and urbanisation was obtained from Central Bureau of Statistics (CBS). To test the change in the counts of food retailers we used negative binomial generalized estimating equations (GEE), with neighbourhoods as the group variable, time as the independent variable and the counts of each type of food retailer as outcome. To account for changes in population density, analyses were adjusted for the number of inhabitants per neighbourhood. We tested effect modification by adding an interaction term for neighbourhood SES and urbanisation to the models.

**Results:**

In Dutch neighbourhoods between 2004 and 2018, a 120 and 35% increase was found in the count of food delivery outlets and restaurants, respectively, and a 24% decrease in count of local food shops. Stratified analyses showed an increase in the availability of supermarkets and convenience stores in the more urbanised and lower SES neighbourhoods, while a decrease was observed in the less urbanised and higher SES neighbourhoods.

**Conclusions:**

We observed considerable changes in the Dutch foodscape. Over a 14 years period, the foodscape changed towards a higher availability of food retailers offering convenience and ready-to-eat foods. These findings can help policy makers aiming to promote a healthier food environment and obesity prevention.

## Background

Current dietary patterns in developed countries are characterized by a great share of energy-dense and high in fat, salt or sugar foods, and high consumption of animal food products and ultra-processed foods. Intakes of fruits and vegetables, fibre and fresh products are generally low and the preparation of home-cooked meals is decreasing [1–4]. Changes in the food environment may be an important driver of unhealthy dietary patterns and their related health consequences such as obesity, type 2 diabetes and cardiovascular diseases [5–7].

The foodscape, i.e., the distribution of food retailers across a determined geographical area, is an important aspect of the food environment [8]. Food environments providing a great amount of opportunities to obtain unhealthy foods may be classified as an ‘obesogenic’ environment, which contributes to population weight gain [9]. Few countries have been able to quantify changes in their foodscape [10–12]. Evidence suggests that some aspects of the foodscape may change relatively quickly, and that the window of change as well as the type of change may differ according to neighbourhood socioeconomic status [10, 12, 13]. Although obesity rates in the Netherlands are lower than in most other countries that are part of the Organisation for Economic Co-operation and Development, the prevalence of obesity has doubled over a period of 20 years [14], and a changing food environment may have contributed to this increase. While research from the Netherlands found evidence for a link between the food environment and dietary patterns and non-communicable diseases [15, 16], no studies have been published to date on how the Dutch foodscape has changed over time. Insight on how the foodscape has changed may be valuable to understand results found in current literature, design new studies as well as policies that aim to facilitate a healthier food environment.

Recent developments such as ongoing urbanisation, differences in levels of affluence, time restraints a growing need for convenience and information technology developments are likely to influence changes in the foodscape. As such, the aim of this study was to analyse if and how the availability of different types of food retailers, adjusted for the number of inhabitants per neighbourhood, has changed between 2004 and 2018, and to explore whether this change (if any) was different according to neighbourhood socioeconomic status (SES) and urbanisation levels.

## Methods

This longitudinal ecological study was conducted across the Netherlands. The units of analysis in this study were administrative neighbourhoods as defined by Central Bureau of Statistics (CBS) of the Netherlands. In order to avoid including non-residential areas -such as natural reserves, neighbourhoods composed mostly of water or big industrial areas-, we included only neighbourhoods with at least 100 inhabitants in the analysis. The median number of inhabitants per neighbourhood in 2004 was 910 (interquartile range = 330–2230) and 2018 it was 985 (interquartile range = 370–2050). Neighbourhood areas in turn, ranged from 0.01 to 131.58 square-kilometres (km^2^) in 2004, and from 0.02 to 130.14 km^2^ in 2018. The number of neighbourhoods analysed each year was not constant as new neighbourhoods emerged and others ceased to exist. Potential changes in some of the neighbourhood boundaries may have occurred. However, CBS annually publishes shapefiles with updated neighbourhood characteristics, as we used these files with updated neighbourhood information for each year, changes on neighbourhood characteristics, such as boundaries, were taken into account in our analysis. In addition, analyses were adjusted for the number of inhabitants per neighbourhood, taking into account changes in the population density.

### Data sources

#### Food environment

The location of all food retailers was determined via geographic coordinates as collected by an independent Dutch company that collects objective data on the Dutch retail landscape (Locatus, https://locatus.com/en/). Since 2004, Locatus systematically performs regular field audits to map the locations and types of stores for commercial purposes; thus, the geographical location of any retailer in the Netherlands is determined. The frequency of field audits varies from once a year - in shopping areas - to once every 2 or 3 years in regions located outside shopping areas. For this study, we included retailers of which the primary activity was to sell food or meals, and excluded other types of retailers that may sell foods as a secondary activity, such as gas stations and drugstores. The validity of Locatus data was tested against a field audit in selected areas across the Netherlands in 2019. This validation study showed the location and classification of grocery stores (e.g., supermarkets, local food shops, green grocers) and food outlets (e.g. restaurants, fast food restaurants) was “good” to "excellent. A kappa value of 0.58 for all food outlets combined was found, showing ‘good’ agreement. However, there were differences in Kappa values across specific food retailer categories. For instance, Kappa values were 0.62 for confectionary stores, 0.92 for supermarkets and 0.95 for fast food retailers. (Canalia 2019, in preparation). Therefore, the Locatus database was considered to be a credible source of data on the locations and classifications of food retailers in the Netherlands. It is worth to note that although Locatus data was considered a secondary data source for this validation study, the data is primarily collected via objective field audits, which is the preferable method for acquisition of geographical data on the location of food retailers.

In the current analysis, food retailers were aggregated into the following categories: local food shops; fast food restaurants; food delivery outlets; restaurants; supermarkets; and convenience stores. Regarding the classification of food retailers, while there are many retailers that have dual purposes (e.g. a full-service restaurant that also do meal delivery), in the Locatus dataset, retailers are classified on the basis of their main purpose. An example is the category ‘food delivery outlets’, which includes solely food retailers that do delivery as their main activity. Similarly, restaurants that provide delivery services are not accounted for in the food delivery category as their main activity is to serve meals at the table. Table 1 provides more detail on the composition of each food retailer category.

**Table 1:** Analysed categories of food retailers

Analytical category	Food retailers composing the analytical category	Definition of food retailers and/or main food products offered by them
Local food shops	Greengrocers	Main provision of potatoes, vegetables and fruit
	Butchery	Main provision of meat and meat products
	Poultry shop	Main provision of poultry
	Bakery	Main provision of bread and pastries. Table service is possible, but this is not be the main store activity
	Fish stores	Main provision of fish, crustaceans and molluscs
Fast food restaurants	Fast food chains and locally owned fast food restaurants	Main provision of mostly deep-fried products that are ready for consumption in few minutes after ordering. Usually there is no table service available.
Food delivery	Food delivery Take away	Main provision of meals that are not consumed in the store, but are collected or delivered
Restaurants	Restaurant	Main provision of meals *a-la-carte*, table service is present. Drinks are only provided in combination with food
	Café-restaurant	Main provision of both drinks and simple meals
	Restaurant in hotels	Main provision of overnight accommodation in combination with an *a-la-carte* restaurant
Supermarket	Supermarket	Store selling a wide range of food and non-food products which are used on a daily bases. Store size should be at least 150 m^2^
Convenience stores	Convenience stores	Same as supermarkets but store size is less than 150 m^2^

#### Neighbourhood level of urbanisation and SES

Based on the density of residential addresses per km^2^, CBS defines 5 levels of urbanisation. Due to the distribution of the urbanisation variable, which had fewer neighbourhoods in the categories indicating highest, high and moderate urbanisation (the highest three categories), we aggregated these categories. By combining these three categories, we obtained a comparable number of observations as in the least urbanised category. Thus, the highest urbanisation category obtained was composed of areas with 1000 addresses or more per km^2^, and the lowest urbanisation category was composed of areas with less than 500 addresses per km^2^.

Information on neighbourhood SES was obtained from the CBS website. Moudon and colleagues (2011), proposed a metric of neighbourhood wealth, which can be derived as a single variable indicating neighbourhood property values, as being a good predictor of individual health status. This metric is considered to be a good proxy of area-based SES in studies that analyse contextual influences on health [17]. Therefore, the average value of residential properties per neighbourhood per year was used as a proxy for neighbourhood SES. Because in 2018 housing prices were not yet available at the time of analysis, the average housing price per neighbourhood in 2017 was used for 2018. In order to obtain a similar variable as the neighbourhood urbanisation variable, the continuous variables for average housing price (neighbourhood SES) were split into quintiles, with the first quintile being composed of the lowest values for average house prices (lowest neighbourhood SES) and the fifth quintile representing the highest values for average house prices (highest neighbourhood SES).

For these analyses, only the top and bottom categories of neighbourhood SES and urbanisation were considered. In the results section we present a table describing the percentage of neighbourhoods in each SES and urbanisation category.

### Statistical analysis

To obtain the counts of food retailers per neighbourhood, we intersected a layer in ArcGIS® containing the location of food retailers to a layer containing the neighbourhood information. Food environment data is typically zero inflated, which usually leads to over-dispersion of the data. Ideally, we would analyse our count data using a zero inflated negative binomial model. However, since we wanted to model ‘time’ as independent variable at the population average level this was not possible, and therefore the generalized estimating equations (GEE) was deemed to be the most suitable. Within the GEE models, both Poisson and Negative binomial models suited our count data. We chose the GEE negative binomial approach because, although it does not account completely for the zero inflated nature of the data (as the zero inflated negative binomial model would), this model does account for over-dispersion. Thus, to test the average change in the count of food retailers from 2004 to 2018, we used negative binomial GEE models with the neighbourhood as the group variable, dummy variables for each year as independent variable and the counts of each food retailer as the outcome in separate models. For these analyses, 2004 was considered the baseline value and yearly changes were compared to 2004.

Because variation in the count of food retailers could reflect variation in population density over time, we adjusted our models for the number of inhabitants per neighbourhood. We also tested whether the average change in the count of food retailers was different according to neighbourhood SES and urbanisation. For this purpose, we tested effect modification by adding an interaction term between ‘year’ and ‘neighbourhood SES’ or ‘urbanisation’ to each model. Given the large number of neighbourhoods, significant interaction was considered with *p* < 0.001. Because we expected a high correlation between SES and urbanisation variables, we analysed the percentage of overlapping neighbourhoods across the categories of neighbourhood SES and urbanisation by running a cross-tabulation table. Also because of this high correlation between SES and urbanisation variables, potentially leading to multi-collinearity, models were run separately, i.e., we did not control for SES in the urbanisation model and vice-versa.

Statistical analyses were conducted in STATA and graphs were produced in RStudio using ggplot2 packages. No smoothing method was applied.

## Results

The total number of neighbourhoods per year included in the analysis ranged from 9956 (in 2004) to 11,751 (in 2018). Some neighbourhoods emerged and others ceased to exist over time, affecting the number of neighbourhood observations per year. All neighbourhood observations over the 14 analysed years summed to a total of 151,150 observations. As in the GEE analysis each neighbourhood observed at least once over the 14 years period counts as one unit of analysis, the final analysed number of neighbourhoods was 15,394. Table 2 shows descriptive neighbourhood statistics at baseline. The maximum number of food retailers per neighbourhood in 2004 was 228, ranging from a maximum number of seven supermarkets to a maximum number of 121 restaurants per neighbourhood. In total, there were 37.8% neighbourhoods with no food retailer present, the amount of ‘empty’ neighbourhoods was higher for highest SES (53.0%) and lowest urbanised neighbourhoods (48%). The median counts of food retailers were mostly zero for the totality of neighbourhoods and also by lowest and highest categories of neighbourhood SES and urbanisation. However, when looking at neighbourhoods with at least one food retailer present, and considering the interquartile ranges, the average counts of all types of food retailers were higher in the low SES and the highly urbanised neighbourhoods than in the high SES and the low urbanised neighbourhoods. Table 3 shows the distribution of neighbourhoods according to neighbourhood SES and urbanisation level. We observed that 75.5% of the lowest SES neighbourhoods were also the highest urbanised neighbourhoods, and 69.3% of all highest SES neighbourhoods were also the least urbanised neighbourhoods.

**Table 2:** Descriptive baseline characteristics of neighbourhoods

	Counts Min-Max	Median (IQR) All neighbourhoods	Neighbourhoods with zero food retailer of each type (%)	Median (IQR) -excluding neighbourhoods with zero food retailers
**Total neighbourhoods in 2004 (*n*** **= 9956)**				
Total counts of food retailers	0–228	1 (0–4)	37.8%	3 (1–7)
Counts of fast food restaurants	0–35	0 (0–1)	65.5%	1 (1–2)
Counts of food delivery places	0–16	0 (0–0)	91.9%	1 (1–2)
Counts of supermarkets	0–7	0 (0–1)	70.3%	1 (1–2)
Counts of local shops	0–39	0 (0–1)	67.2%	2 (1–4)
Counts of restaurants	0–121	0 (0–1)	60.6%	1 (1–3)
Counts of convenience stores	0–10	0 (0–0)	90.4%	1 (1–1)
Inhabitants per neighbourhood	100–27,500	910 (330–2230)	–	–
**Lowest neighbourhood SES in 2004 (*n*** **= 1936)**				
Total counts of food retailers	0–157	3 (0–8)	25.2%	5 (2–11)
Counts of fast food restaurants	0–24	1 (0–2)	46.6%	2 (1–3)
Counts of food delivery places	0–8	0 (0–0)	82.3%	1 (1–2)
Counts of supermarkets	0–7	0 (0–1)	54.7%	1 (1–2)
Counts of local shops	0–39	0 (0–2)	50.5%	2 (1–5)
Counts of restaurants	0–77	0 (0–1)	55.5%	2 (1–3)
Counts of convenience stores	0–10	0 (0–0)	81.6%	1 (1–2)
Inhabitants per neighbourhood	100–27,500	1645 (760–3140)	–	–
**Highest neighbourhood SES in 2004 (*n*** **= 1875)**				
Total counts of food retailers	0–170	0 (0–2)	53.0%	2 (1–3)
Counts of fast food restaurants	0–19	0 (0–0)	87.9%	1 (1–1)
Counts of food delivery places	0–8	0 (0–0)	98.3%	1 (1–2)
Counts of supermarkets	0–3	0 (0–0)	90.2%	1 (1–1)
Counts of local shops	0–16	0 (0–0)	87.4%	1 (1–2)
Counts of restaurants	0–98	0 (0–1)	67.5%	1 (1–2)
Counts of convenience stores	0–3	0 (0–0)	94.9%	1 (1–1)
Inhabitants per neighbourhood	100–11,430	340 (200–680)	–	
**Lowest neighbourhood urbanisation in 2004 (*n*** **= 4554)**				
Total counts of food retailers	0–55	1 (0–2)	48.8%	2 (1–4)
Counts of fast food restaurants	0–7	0 (0–0)	81.8%	1 (1–2)
Counts of food delivery places	0–1	0 (0–0)	99.1%	1 (1–1)
Counts of supermarkets	0–3	0 (0–0)	80.4%	1 (1–1)
Counts of local shops	0–8	0 (0–0)	79.4%	1 (1–2)
Counts of restaurants	0–34	0 (0–1)	66.9%	1 (1–2)
Counts of convenience stores	0–3	0 (0–0)	93.7%	1 (1–1)
Inhabitants per neighbourhood	100–5220	390 (200–820)	–	–
**Highest neighbourhood urbanisation in 2004 (*n*** **= 3576)**				
Total counts of food retailers	0–228	3 (0–8)	28.4%	5 (2–12)
Counts of fast food restaurants	0–35	1 (0–2)	47.1%	2 (1–3)
Counts of food delivery places	0–16	0 (0–0)	81.0%	1 (1–2)
Counts of supermarkets	0–7	0 (0–1)	58.0%	1 (1–2)
Counts of local shops	0–39	0 (0–3)	51.3%	3 (1–5)
Counts of restaurants	0–121	0 (0–2)	56.2%	2 (1–4)
Counts of convenience stores	0–10	0 (0–0)	84.3%	1 (1–2)
Inhabitants per neighbourhood	100–27,500	2010 (1070–13,630)	–	–

SES: socioeconomic status; IQR: interquartile range

**Table 3:** Distribution of neighbourhoods according to neighbourhood socioeconomic status (SES) and urbanisation levels

Neighbourhood SES		Urbanisation				
		Highest ^a^				Lowest
		1	2	3	4	5
Lowest	1 (*n* = 27,877 ^b^)	**26.7%**	**33.2%**	**15.6%**	8.7%	16.0%
	2 (n = 27,735 ^b^)	15.2%	25.6%	17.1%	16.0%	26.2%
	3 (n = 27,644 ^b^)	7.8%	17.9%	16.6%	19.1%	38.6%
	4 (n = 27,511 ^b^)	5.5%	10.1%	12.6%	18.0%	53.8%
Highest	5 (n = 27,523 ^b^)	3.9%	5.9%	7.2%	13.6%	**69.3%**

^a^The highest urbanisation category is composed of the highest 3 urbanisation levels as defined by the Central Bureau of Statistics (CBS). Values in bold represent the overlap between lowest and highest categories of neighbourhood SES and urbanisation

^b^Number of observations for neighbourhood SES across the 14 years period, this includes observations for neighbourhoods that ceased to exist or were created during the study period. For all the study years, lowest urbanisation category is composed of neighbourhoods with < 500 addresses/km^2^; highest urbanisation category is composed of neighbourhoods with ≥1000 addresses/km^2^. Lowest SES category is composed of neighbourhoods with an average house value ranging from EUR 25,000 to EUR 191,000; Highest SES category is composed of neighbourhoods with an average house value of EUR 209,000 or higher

**Fig. 1:**
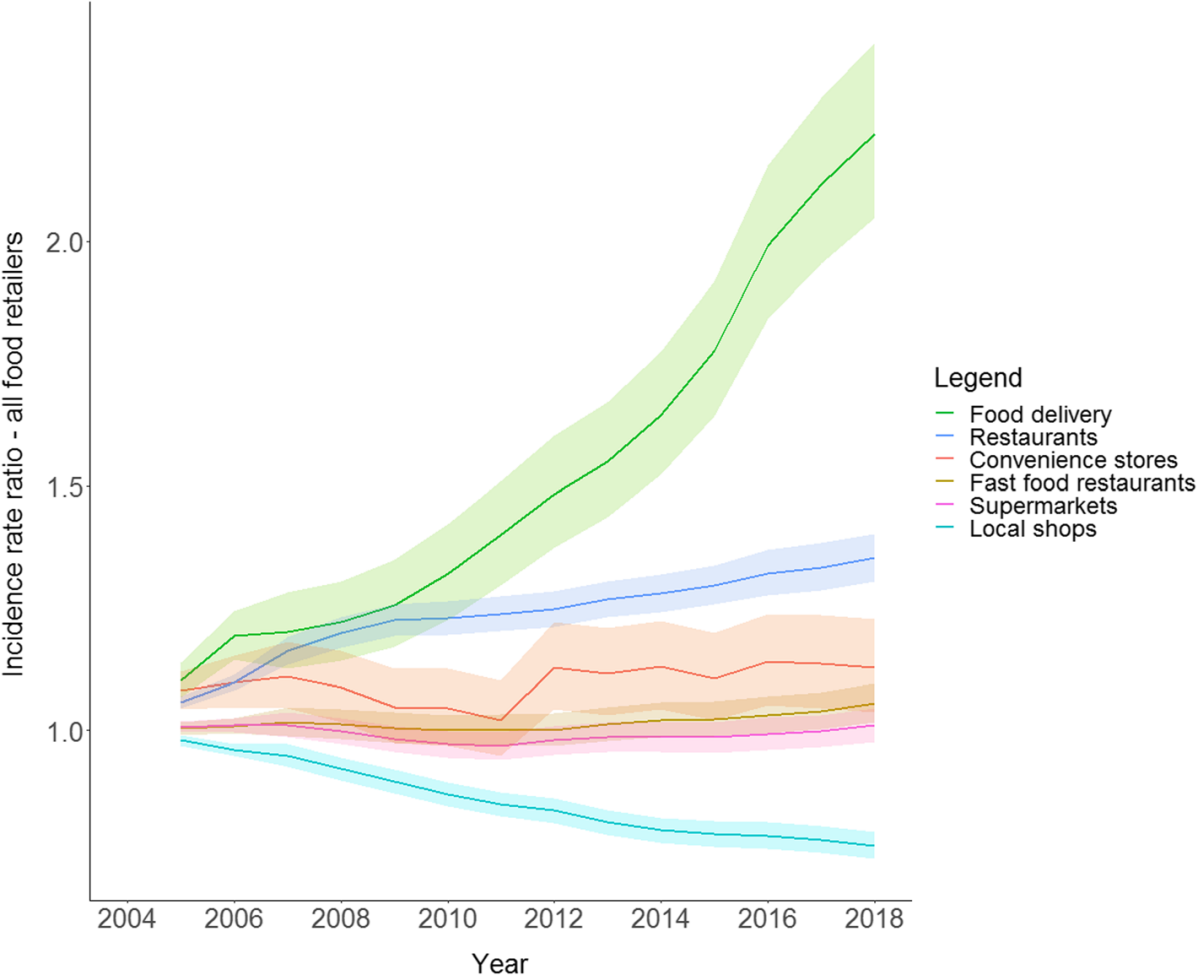
Incidence rate ratio and 95% confidence interval for average change in the neighbourhood counts of food retailers in the Netherlands. Coefficients were derived from negative binomial generalized estimating equations (GEE) analysis. Analyses were adjusted for the number of inhabitants per neighbourhoods

Figure 1 shows the average change in the counts per neighbourhood of the various types of food retailers in the Netherlands. The biggest changes were observed for food delivery places, restaurants and local shops. Neighbourhoods in 2018, as compared to 2004, had a 120% increase in the count of food delivery outlets (incidence rate ratio (IRR) = 2.22, 95% confidence interval (CI) = 2.04–2.41); 35% increase in the counts of restaurants (IRR = 1.35, 95% CI = 1.30–1.40); and 24% decrease in count of local shops (IRR = 0.76, 95% CI = 0.74–0.79). A weaker increase was observed for convenience stores (IRR = 1.13, 95% CI = 1.04–1.23) and fast food restaurants (IRR = 1.06, 95% CI = 1.02–1.10) in 2018 as compared to 2004. For counts of supermarkets, no significant change was observed in 2018 as compared to 2004 (IRR = 1.01, 95% CI = 0.98–1.04).

**Fig. 2:**
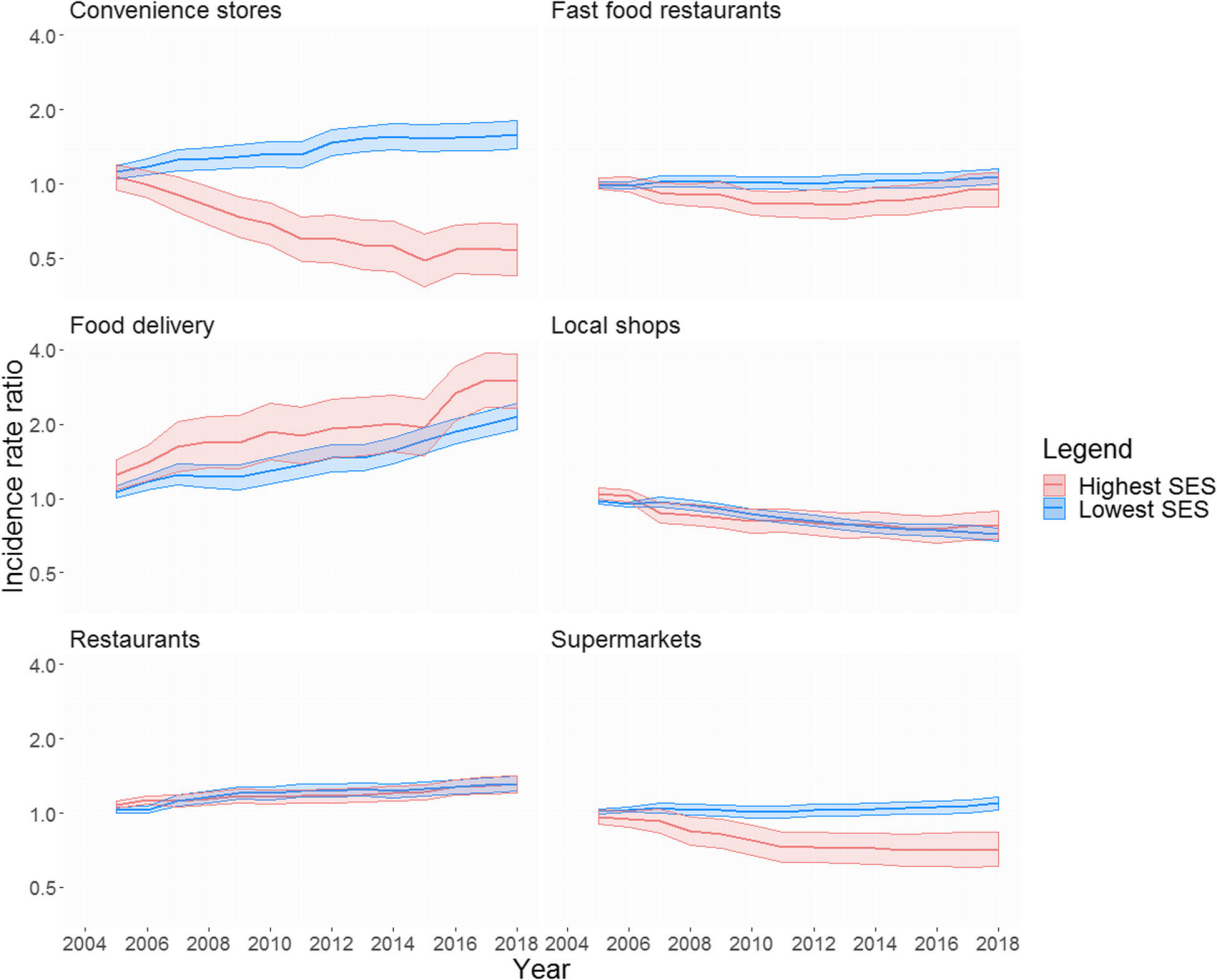
Incidence rate ratio and 95% confidence interval for average change in the counts of food retailers across neighbourhoods with high and low socio-economic status in the Netherlands (*p* value for interaction < 0.001). Coefficients were derived from negative binomial generalized estimating equations (GEE) analysis. Analyses were adjusted for number of inhabitants per neighbourhood

Significant effect modification (*p* < 0.001) was observed for a change in the availability of convenience stores and supermarkets across lowest and highest neighbourhood SES and urbanisation. Figure 2 shows stratified analyses by neighbourhood SES. The count of both convenience stores and supermarkets was higher in the lowest SES neighbourhoods over the entire study period, with the average counts of convenience stores increasing 58% (IRR = 1.58, 95%CI = 1.38–1.80) in the lowest, and decreasing 46% (IRR = 0.54, 95%CI = 0.43–0.68) in the highest SES neighbourhood strata in 2018 as compared to 2004. For supermarkets, an increase of 10% and a decrease of 29% was observed for lowest (IRR = 1.10, 95%CI = 1.03–1.17) and highest (IRR = 0.71, 95%CI = 0.61–0.84) SES neighbourhood, respectively. Food delivery places increased in both lowest (IRR = 2.15, 95%CI = 1.90–2.44) and highest (IRR = 3.00, 95%CI = 2.32–3.87) SES neighbourhoods. Although the average counts of food delivery outlets were higher for the highest SES neighbourhoods over the entire study period, no effect modification by neighbourhood SES was found. Local shops decreased and restaurants increased from 2004 to 2018, but there were no differences across lowest and highest SES neighbourhoods and the effect sizes across both neighbourhood types were very similar: local shops had a 29 and 22% decrease for lowest and highest SES neighbourhood respectively, and restaurants had approximately 30% increase in both neighbourhood types. While average count of fast food restaurants was higher for the lowest SES neighbourhood over the study period, no significant change was observed in both lowest and highest SES neighbourhood from 2004 to 2018.

**Fig. 3:**
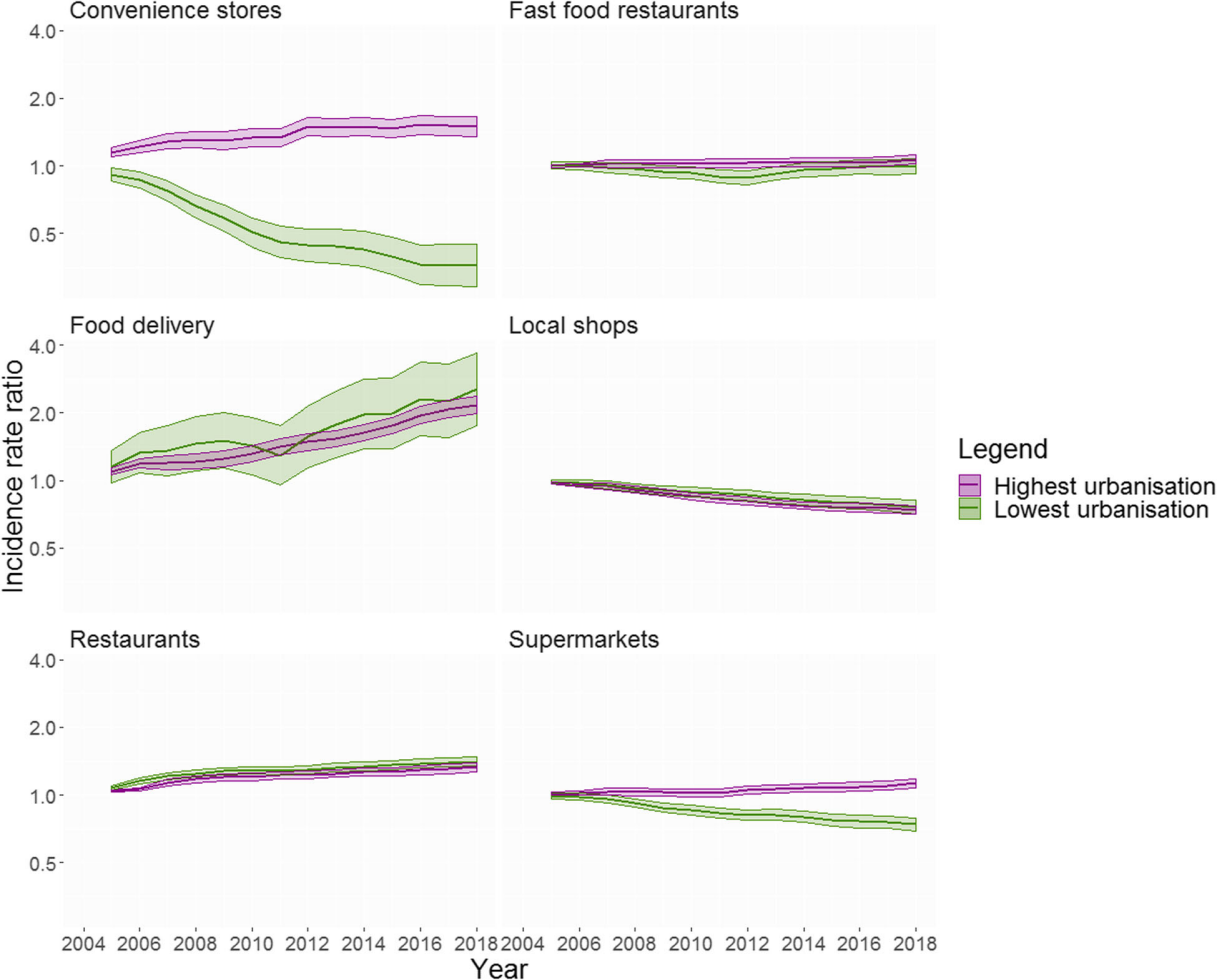
Incidence rate ratios and 95% confidence intervals for average changes in the counts of food retailers across neighbourhoods with high and low urbanisation levels in the Netherlands (p value for interaction < 0.001). Coefficients were derived from negative binomial generalized estimating equations (GEE) analysis. Analyses were adjusted for number of inhabitants per neighbourhood

Figure 3 shows the results of stratified analyses by urbanisation level. Similar to the analysis stratified by neighbourhood SES, significant effect modification (*p* < 0.001) was observed for a change in the availability of convenience stores and supermarkets across lowest and highest levels of urbanisation. In 2018, as compared to 2004, counts of convenience stores increased by 50% (IRR = 1.50, 95% CI = 1.35–1.66) and supermarkets increased by 13% (IRR = 1.13, 95% CI = 1.08–1.18) in the highest urbanised neighbourhoods. In the lowest urbanised neighbourhoods, convenience stores decreased 64% (IRR = 0.36, 95% CI = 0.29–0.45) and supermarkets decreased 26% (IRR = 0.74, 95% CI = 0.69–0.79). Food delivery places and restaurants increased in both levels of urbanisation. Food delivery outlets had a 154% increase in the lowest (IRR = 2.54, 95% CI = 1.75–3.68) and a 116% increase in the highest urbanised neighbourhoods (IRR = 2.16, 95% CI = 1.98–2.36). Restaurants increased 41% in the lowest (IRR = 1.41, 95% CI = 1.34–1.48) and 33% in the highest urbanised neighbourhoods (IRR = 1.33, 95% CI = 1.26–1.40). For fast food restaurants, while no significant effect modification was found, in the stratified analysis a small increase of 7% was observed in the highest urbanised neighbourhoods (IRR = 1.07, 95% CI = 1.02–1.12). Similarly, no differences across levels of urbanisation were observed for local shops as a decrease of about 25% was observed for both lowest (IRR = 0.76, 95%CI = 0.71–0.81) and highest (IRR = 0.74, 95% CI = 0.70–0.77) urbanisation levels.

## Discussion

In this study we analysed changes in the Dutch foodscape over the years 2004 to 2018 and explored whether these changes were different according to neighbourhood SES and levels of urbanisation. Independent of neighbourhood SES and urbanisation, we found a remarkable increase in the availability of food delivery outlets, restaurants and to a lesser extent in fast food restaurants. A general decrease was observed in the availability of local food shops. While in the general analysis, the availability of supermarkets and convenience stores did not seem to change, this apparent null effect was most likely due to effect modification by neighbourhood SES and urbanisation levels. Indeed, in the stratified analysis, more urbanised as well as lower SES neighbourhoods showed an increase in the availability of supermarkets and convenience stores, while a decrease was observed in the less urbanised and higher SES neighbourhoods.

Not many studies to date have analysed trends in the foodscape. In addition, previous studies investigated different kinds of food retailers, used different classifications, or are from countries depicting a different foodscape than in the Netherlands [11, 12]. Nonetheless, a study from the UK found a general increase of 45% in the availability of takeaway outlets over an 18 years period, while a stronger increase of 120% was observed in our study for food delivery places, which included takeaway outlets. Also, they found an increase in the availability of supermarkets, while in our study the availability of supermarkets remained constant in the general analysis [12]. Similar to our findings, a study from the US observed an increase in the availability of restaurants and fast food restaurants and a decrease in the availability of independent grocery stores, while the availability of supermarkets remained relatively constant. Also, they observed an increase in the availability of bakeries, while in our study, bakeries were part of the local shop category, which generally decreased over time [11]. These general trends of increasing availability of food delivery outlets and restaurants and the decreased availability of local shops are in line with previous changes in dietary patterns of populations that shifted towards fewer home cooked meals and more ready-to-eat meals and eating-out [1, 3, 4]. It is worth highlighting that in our analyses, only food retailers whose main activity was meal delivery or take away were accounted for in the ‘food delivery’ category. However, it is well known that a major part of other type of food retailers such as restaurants and pizzerias, besides offering on-site table service, also offer food delivery, either by their own or by making use of web/mobile applications. Therefore, the steep increase in the availability of food delivery places observed in this study is likely to be even higher. This scenario may potently lead to less healthy diets as eating out of home has been associated with poorer diets and weight gain, while eating home-cooked meals have been associated with better quality diets [18–20].

Disparities in the neighbourhood food environment and access to food have been documented before [21, 22], and there is convincing evidence from cross-sectional studies, especially from the US, that residents of lower SES neighbourhoods have higher access to food retailers selling unhealthy foods such as fast foods, and lower access to food retailers selling healthier options [22]. We found that availability of fast food restaurants was higher in the lowest SES neighbourhood over the entire study period. However, there was no significant difference in change in the availability of fast food restaurants and food delivery outlets across neighbourhood SES and urbanisation categories. This suggests that there was no evidence for increasing disparities in access to fast food in the Dutch foodscape in the analysed period. This is in contrast with a study from the UK which found a greater increase in the availability of fast food restaurants and food delivery outlets in lower SES areas, enlarging socioeconomic differences in the access to food retailers [12]. Evidence from the US showed that residents of lower SES areas had the greater access to fast food retailers over a 40 year-period, however, since more affluent areas had a more steep increase in access to fast food retailers, differences between different strata of neighbourhood SES decreased over time [11]. A significant change, however, was found in our study for an increase in the availability of convenience stores and supermarkets in the lowest SES neighbourhoods.

A tendency towards an increase in access to supermarkets across lower SES areas was also found in previous research [11, 12]. This may be due to the fact that it is more expensive for owners to open and maintain stores in more affluent neighbourhoods where the price of real estate is higher. Supermarkets have been mostly considered a source of healthier food options and convenience stores have been considered a source of less healthy foods [23]. However, it is worth mentioning that in the Netherlands convenience stores are mostly small-scale supermarkets found mainly in train stations and shopping areas. These “mini-supermarkets” also offer a range of healthy products such as ready-to-eat salads and chopped fruits to take away. Therefore, based only on the location of food retailers (i.e., community food retail environment), we cannot conclude that lowest SES neighbourhoods developed a less healthy foodscape over this 14-year period. Insight into the consumer food retail environment (i.e., what is sold in each type of food outlet, and how the in-store offer of foods and drinks has changed over time) would therefore be of added value. It also needs to be noted that there was considerable overlap between the neighbourhood SES and urbanisation variables. That is, low SES neighbourhoods were often highly urbanised neighbourhoods, and high SES neighbourhoods were often low urbanised neighbourhoods. Observed SES-disparities may thus be attributable to urbanisation-disparities and vice versa, being hard to disentangle whether the differences found in our study are due to urban-rural or SES differences.

This study has some limitations. Although other relevant dimensions of the food environment such as opening times, quality and price of the foods offered in stores have probably changed over time [24], we had only information on the location of stores, and therefore analysed the foodscape solely in terms of type and geographic availability of food retailers. By using negative binomial GEE models, we were able to account for the over-dispersion of our count variables, which is an usual consequence of the a high number of zeros. However, this model does not account completely for the zero inflated nature of the data. A potential consequence of not accounting for zero inflation in the data is that statistically significant findings may be missed [25]. Therefore, although unlikely due our large sample of neighbourhoods, it is possible that we were unable to identify all the potential significant associations. We highlight the innovative aspect of this study as being the first to analyse the Dutch foodscape. As most previous studies could only analyse a few food retailers, in a limited area and using telephone or governmental directories data [10–12], it is a strength of our study that we analysed a wide range of food retailers, including food delivery outlets, using high quality objectively measured data across the entire country.

## Conclusions

In conclusion, we observed important changes in the Dutch foodscape over a 14 years period. In general, the availability of food delivery outlets, restaurants and - to a lesser extent - fast food restaurants increased, while the availability of local food shops such as greengrocers, butchers and bakeries decreased. The only considerable differences across levels of neighbourhood SES and urbanisation were observed for convenience stores and supermarkets. Therefore, although disparities in the Dutch foodscape may not be so evident over the analysed period, in general, the foodscape has changed towards a higher availability of food retailers offering convenience and ready-to-eat foods. Similar, but context-dependent, changes may have taken place in other high-income countries as well, contributing to the global obesity epidemic. These findings can help policy makers aiming to promote a healthier food environment and obesity prevention. Future studies could link observed changes in the foodscape to national surveillance data on dietary behaviours and non-communicable disease trends.

## Supplementary information

**Additional file 1: Supplementary Table 1.** Incidence rate ratio and 95% confidence interval as derived from negative binomial generalized estimating equations (GEE) analysis. Coefficients represent the average change in the neighbourhood counts of fast food retailers in the Netherlands. Coefficients are presented for the totality of neighbourhoods, as well as according to neighbourhood socio-economic status (SES) and urbanisation levels. **Supplementary Table 2.** Incidence rate ratio and 95% confidence interval as derived from negative binomial generalized estimating equations (GEE) analysis. Coefficients represent the average change in the neighbourhood counts of food delivery outlets in the Netherlands. Coefficients are presented for the totality of neighbourhoods, as well as according to neighbourhood socio-economic status (SES) and urbanisation levels. **Supplementary Table 3.** Incidence rate ratio and 95% confidence interval as derived from negative binomial generalized estimating equations (GEE) analysis. Coefficients represent the average change in the neighbourhood counts of supermarkets in the Netherlands. Coefficients are presented for the totality of neighbourhoods, as well as according to neighbourhood socio-economic status (SES) and urbanisation levels. **Supplementary Table 4.** Incidence rate ratio and 95% confidence interval as derived from negative binomial generalized estimating equations (GEE) analysis. Coefficients represent the average change in the neighbourhood counts of local food shops in the Netherlands. Coefficients are presented for the totality of neighbourhoods, as well as according to neighbourhood socio-economic status (SES) and urbanisation levels. **Supplementary Table 5.** Incidence rate ratio and 95% confidence interval as derived from negative binomial generalized estimating equations. Coefficients represent the average change in the neighbourhood counts of restaurants in the Netherlands. Coefficients are presented for the totality of neighbourhoods, as well as according to neighbourhood socio-economic status (SES) and urbanisation levels. (GEE) analysis. **Supplementary Table 6.** Incidence rate ratio and 95% confidence interval as derived from negative binomial generalized estimating equations (GEE) analysis. Coefficients represent the average change in the neighbourhood counts of convenience stores in the Netherlands. Coefficients are presented for the totality of neighbourhoods, as well as according to neighbourhood socio-economic status (SES) and urbanisation levels.

## Data Availability

The data that support the findings of this study are available from Locatus® but restrictions apply to the availability of these data, which were used under license for the current study, and so are not publicly available. Data are however available from the authors upon reasonable request and with permission of Locatus®.

## Abbreviations

SES: Socioeconomic status
CBS: Central Bureau of Statistics
GEE: Generalized estimating equations
IRR: Incidence rate ratio
km^2^: Square-kilometres
95%CI: 95% confidence intervals
