# Supplementary material for: Recent changes in the Dutch foodscape: socioeconomic and urban-rural differences
Source: Int J Behav Nutr Phys Act. 2020 Mar 20;17:43. doi: 10.1186/s12966-020-00944-5 (PMC7083034; doi:10.1186/s12966-020-00944-5)
Supplement: Supplementary file 1 — Additional file 1: Supplementary Table 1. Incidence rate ratio and 95% confidence interval as derived from negative binomial generalized estimating equations (GEE) analysis. Coefficients represent the average change in the neighbourhood counts of fast food retailers in the Netherlands. Coefficients are presented for the totality of neighbourhoods, as well as according to neighbourhood socio-economic status (SES) and urbanisation levels. Supplementary Table 2. Incidence rate ratio and 95% confidence interval as derived from negative binomial generalized estimating equations (GEE) analysis. Coefficients represent the average change in the neighbourhood counts of food delivery outlets in the Netherlands. Coefficients are presented for the totality of neighbourhoods, as well as according to neighbourhood socio-economic status (SES) and urbanisation levels. Supplementary Table 3. Incidence rate ratio and 95% confidence interval as derived from negative binomial generalized estimating equations (GEE) analysis. Coefficients represent the average change in the neighbourhood counts of supermarkets in the Netherlands. Coefficients are presented for the totality of neighbourhoods, as well as according to neighbourhood socio-economic status (SES) and urbanisation levels. Supplementary Table 4. Incidence rate ratio and 95% confidence interval as derived from negative binomial generalized estimating equations (GEE) analysis. Coefficients represent the average change in the neighbourhood counts of local food shops in the Netherlands. Coefficients are presented for the totality of neighbourhoods, as well as according to neighbourhood socio-economic status (SES) and urbanisation levels. Supplementary Table 5. Incidence rate ratio and 95% confidence interval as derived from negative binomial generalized estimating equations. Coefficients represent the average change in the neighbourhood counts of restaurants in the Netherlands. Coefficients are presented for the totality of neighbour [file 12966_2020_944_MOESM1_ESM.docx]

Supplementary Table 1. Incidence rate ratio and 95% confidence interval as derived from negative binomial generalized estimating equations (GEE) analysis. Coefficients represent the average change in the neighbourhood counts of fast food retailers in the Netherlands. Coefficients are presented for the totality of neighbourhoods, as well as according to neighbourhood socio-economic status (SES) and urbanisation levels.

|  |  | All neighbourhoods | Lowest SES neighbourhoods | Highest SES neighbourhoods | Lowest urbanisation | Highest urbanisation |
| --- | --- | --- | --- | --- | --- | --- |
| Food retailer category | Year | Coefficient | Coefficient | Coefficient | Coefficient | Coefficient |
|  |  | (95% CI) | (95% CI) | (95% CI) | (95% CI) | (95% CI) |
| Fast food restaurants | 2004 | Reference | Reference | Reference | Reference | Reference |
|  | 2005 | 1.00 (0.99 - 1.02) | 0.99 (0.96 - 1.02) | 1.00 (0.95 - 1.06) | 1.01 (0.97 - 1.04) | 1.00 (0.98 - 1.02) |
|  | 2006 | 1.01 (0.99 - 1.03) | 0.99 (0.96 - 1.02) | 0.99 (0.93 - 1.06) | 1.00 (0.96 - 1.05) | 1.01 (0.99 - 1.03) |
|  | 2007 | 1.02 (0.99 - 1.05) | 1.02 (0.97 - 1.07) | 0.92 (0.83 - 1.01) | 0.98 (0.93 - 1.04) | 1.02 (0.98 - 1.07) |
|  | 2008 | 1.01 (0.98 - 1.04) | 1.02 (0.97 - 1.08) | 0.90 (0.81 - 1.00) | 0.97 (0.92 - 1.02) | 1.02 (0.98 - 1.06) |
|  | 2009 | 1.01 (0.97 - 1.04) | 1.02 (0.96 - 1.08) | 0.90 (0.80 - 1.02) | 0.94 (0.89 - 1.00) | 1.02 (0.98 - 1.07) |
|  | 2010 | 1.00 (0.97 - 1.03) | 1.01 (0.96 - 1.07) | 0.83 (0.74 - 0.93) | 0.93 (0.87 - 0.99) | 1.02 (0.98 - 1.06) |
|  | 2011 | 1.00 (0.97 - 1.03) | 1.01 (0.95 - 1.07) | 0.83 (0.73 - 0.93) | 0.90 (0.84 - 0.96) | 1.03 (0.98 - 1.07) |
|  | 2012 | 1.00 (0.97 - 1.03) | 1.00 (0.94 - 1.07) | 0.83 (0.73 - 0.94) | 0.88 (0.82 - 0.95) | 1.03 (0.98 - 1.07) |
|  | 2013 | 1.01 (0.98 - 1.05) | 1.02 (0.96 - 1.09) | 0.82 (0.72 - 0.93) | 0.93 (0.86 - 0.99) | 1.03 (0.99 - 1.08) |
|  | 2014 | 1.02 (0.99 - 1.06) | 1.03 (0.97 - 1.10) | 0.85 (0.75 - 0.97) | 0.96 (0.89 - 1.03) | 1.04 (0.99 - 1.08) |
|  | 2015 | 1.02 (0.99 - 1.06) | 1.03 (0.96 - 1.10) | 0.86 (0.75 - 0.98) | 0.97 (0.90 - 1.05) | 1.04 (0.99 - 1.08) |
|  | 2016 | 1.03 (0.99 - 1.07) | 1.03 (0.97 - 1.10) | 0.89 (0.78 - 1.02) | 0.99 (0.92 - 1.07) | 1.03 (0.99 - 1.08) |
|  | 2017 | 1.04 (1.00 - 1.08) | 1.05 (0.98 - 1.13) | 0.94 (0.81 - 1.10) | 0.99 (0.92 - 1.07) | 1.05 (1.00 - 1.10) |
|  | 2018 | 1.06 (1.02 - 1.10) | 1.07 (1.00 - 1.15) | 0.94 (0.80 - 1.11) | 1.00 (0.93 - 1.08) | 1.07 (1.02 - 1.12) |

Model was adjusted for number of inhabitants per neighbourhood.

Supplementary Table 2. Incidence rate ratio and 95% confidence interval as derived from negative binomial generalized estimating equations (GEE) analysis. Coefficients represent the average change in the neighbourhood counts of food delivery outlets in the Netherlands. Coefficients are presented for the totality of neighbourhoods, as well as according to neighbourhood socio-economic status (SES) and urbanisation levels.

|  |  | All neighbourhoods | Lowest SES neighbourhoods | Highest SES neighbourhoods | Lowest urbanisation | Highest urbanisation |
| --- | --- | --- | --- | --- | --- | --- |
| Food retailer category | Year | Coefficient | Coefficient | Coefficient | Coefficient | Coefficient |
|  |  | (95% CI) | (95% CI) | (95% CI) | (95% CI) | (95% CI) |
| Food delivery outlets | 2004 | Reference | Reference | Reference | Reference | Reference |
|  | 2005 | 1.10 (1.07 - 1.14) | 1.06 (1.00 - 1.12) | 1.25 (1.08 - 1.44) | 1.15 (0.97 - 1.36) | 1.10 (1.06 - 1.14) |
|  | 2006 | 1.19 (1.14 - 1.24) | 1.16 (1.09 - 1.25) | 1.39 (1.18 - 1.64) | 1.32 (1.08 - 1.63) | 1.19 (1.13 - 1.24) |
|  | 2007 | 1.20 (1.13 - 1.28) | 1.25 (1.13 - 1.38) | 1.62 (1.28 - 2.05) | 1.36 (1.05 - 1.76) | 1.19 (1.11 - 1.28) |
|  | 2008 | 1.22 (1.14 - 1.30) | 1.23 (1.10 - 1.36) | 1.70 (1.33 - 2.16) | 1.46 (1.10 - 1.93) | 1.22 (1.13 - 1.31) |
|  | 2009 | 1.26 (1.17 - 1.35) | 1.22 (1.09 - 1.37) | 1.69 (1.31 - 2.17) | 1.51 (1.13 - 2.01) | 1.25 (1.16 - 1.35) |
|  | 2010 | 1.32 (1.23 - 1.42) | 1.30 (1.15 - 1.46) | 1.86 (1.43 - 2.42) | 1.43 (1.06 - 1.91) | 1.32 (1.21 - 1.43) |
|  | 2011 | 1.40 (1.30 - 1.51) | 1.38 (1.21 - 1.56) | 1.80 (1.38 - 2.35) | 1.29 (0.95 - 1.75) | 1.41 (1.30 - 1.53) |
|  | 2012 | 1.48 (1.37 - 1.60) | 1.46 (1.29 - 1.66) | 1.92 (1.47 - 2.52) | 1.56 (1.13 - 2.15) | 1.48 (1.36 - 1.62) |
|  | 2013 | 1.55 (1.44 - 1.67) | 1.47 (1.30 - 1.66) | 1.96 (1.50 - 2.57) | 1.78 (1.26 - 2.50) | 1.54 (1.42 - 1.67) |
|  | 2014 | 1.64 (1.52 - 1.77) | 1.56 (1.38 - 1.76) | 2.01 (1.55 - 2.61) | 1.97 (1.38 - 2.82) | 1.63 (1.50 - 1.77) |
|  | 2015 | 1.78 (1.64 - 1.92) | 1.72 (1.53 - 1.94) | 1.93 (1.49 - 2.51) | 1.99 (1.39 - 2.85) | 1.76 (1.61 - 1.91) |
|  | 2016 | 1.99 (1.84 - 2.16) | 1.87 (1.66 - 2.11) | 2.68 (2.08 - 3.45) | 2.31 (1.59 - 3.35) | 1.95 (1.79 - 2.13) |
|  | 2017 | 2.12 (1.96 - 2.29) | 2.00 (1.77 - 2.26) | 3.01 (2.34 - 3.88) | 2.26 (1.55 - 3.29) | 2.07 (1.90 - 2.26) |
|  | 2018 | 2.22 (2 .05 - 2.41) | 2.15 (1.90 - 2.44) | 3.00 (2.32 - 3.87) | 2.54 (1.75 - 3.68) | 2.16 (1.98 - 2.36) |

Model was adjusted for number of inhabitants per neighbourhood.

Supplementary Table 3. Incidence rate ratio and 95% confidence interval as derived from negative binomial generalized estimating equations (GEE) analysis. Coefficients represent the average change in the neighbourhood counts of supermarkets in the Netherlands. Coefficients are presented for the totality of neighbourhoods, as well as according to neighbourhood socio-economic status (SES) and urbanisation levels.

|  |  | All neighbourhoods | Lowest SES neighbourhoods | Highest SES neighbourhoods | Lowest urbanisation | Highest urbanisation |
| --- | --- | --- | --- | --- | --- | --- |
| Food retailer category | Year | Coefficient | Coefficient | Coefficient | Coefficient | Coefficient |
|  |  | (95% CI) | (95% CI) | (95% CI) | (95% CI) | (95% CI) |
| Supermarkets | 2004 | Reference | Reference | Reference | Reference | Reference |
|  | 2005 | 1.01 (1.00 - 1.02) | 1.02 (0.99 - 1.04) | 0.96 (0.90 - 1.03) | 0.99 (0.97 - 1.02) | 1.01 (1.00 - 1.03) |
|  | 2006 | 1.01 (1.00 - 1. 02) | 1.03 (1.01 - 1.06) | 0.94 (0.88 - 1.02) | 0.98 (0.95 - 1.02) | 1.03 (1.01 - 1.04) |
|  | 2007 | 1.01 (0.99 - 1. 04) | 1.05 (1.00 - 1.10) | 0.93 (0.83 - 1.03) | 0.96 (0.92 - 1.00) | 1.04 (1.00 - 1.08) |
|  | 2008 | 1.00 (0.97 - 1.03) | 1.03 (0.98 - 1.09) | 0.85 (0.74 - 0.96) | 0.92 (0.88 - 0.96) | 1.03 (0.99 - 1.07) |
|  | 2009 | 0.98 (0.96 - 1.01) | 1.03 (0.97 - 1.08) | 0.82 (0.72 - 0.94) | 0.88 (0.84 - 0.92) | 1.03 (0.99 - 1.07) |
|  | 2010 | 0.97 (0.94 - 1.00) | 1.01 (0.96 - 1.06) | 0.78 (0.68 - 0.89) | 0.86 (0.82 - 0.90) | 1.02 (0.98 - 1.06) |
|  | 2011 | 0.97 (1.04 - 1.00) | 1.01 (0.95 - 1.07) | 0.72 (0.63 - 0.83) | 0.83 (0.79 - 0.88) | 1.03 (0.98 - 1.07) |
|  | 2012 | 0.98 (0.95 - 1.01) | 1.03 (0.97 - 1.09) | 0.73 (0.63 - 0.83) | 0.81 (0.77 - 0.86) | 1.05 (1.01 - 1.10) |
|  | 2013 | 0.99 (0.96 - 1.02) | 1.03 (0.98 - 1.09) | 0.72 (0.62 - 0.83) | 0.82 (0.77 - 0.87) | 1.06 (1.02 - 1.11) |
|  | 2014 | 0.99 (0.96 - 1.02) | 1.04 (0.98 - 1.10) | 0.72 (0.62 - 0.83) | 0.80 (0.76 - 0.85) | 1.07 (1.03 - 1.12) |
|  | 2015 | 0.99 (0.95 - 1.02) | 1.05 (0.99 - 1.11) | 0.71 (0.61 - 0.82) | 0.77 (0.73 - 0.82) | 1.08 (1.03 - 1.13) |
|  | 2016 | 0.99 (0.96 - 1.03) | 1.06 (0.99 - 1.12) | 0.71 (0.61 - 0.83) | 0.76 (0.72 - 0.81) | 1.09 (1.04 - 1.14) |
|  | 2017 | 1.00 (0.97 - 1.03) | 1.07 (1.00 - 1.13) | 0.71 (0.60 - 0.83) | 0.76 (0.71 - 0.81) | 1.10 (1.05 - 1.15) |
|  | 2018 | 1.01 (0.98 - 1.04) | 1.10 (1.03 - 1.17) | 0.71 (0.61 - 0.84) | 0.74 (0.69 - 0.79) | 1.13 (1.08 - 1.18) |

Model was adjusted for number of inhabitants per neighbourhood.

Supplementary Table 4. Incidence rate ratio and 95% confidence interval as derived from negative binomial generalized estimating equations (GEE) analysis. Coefficients represent the average change in the neighbourhood counts of local food shops in the Netherlands. Coefficients are presented for the totality of neighbourhoods, as well as according to neighbourhood socio-economic status (SES) and urbanisation levels.

|  |  | All neighbourhoods | Lowest SES neighbourhoods | Highest SES neighbourhoods | Lowest urbanisation | Highest urbanisation |
| --- | --- | --- | --- | --- | --- | --- |
| Food retailer category | Year | Coefficient | Coefficient | Coefficient | Coefficient | Coefficient |
|  |  | (95% CI) | (95% CI) | (95% CI) | (95% CI) | (95% CI) |
| Local food shops | 2004 | Reference | Reference | Reference | Reference | Reference |
|  | 2005 | 0.98 (0.97 - 0.99) | 0.98 (0.95 - 1.00) | 1.05 (0.99 - 1.10) | 0.99 (0.97 - 1.01) | 0.97 (0.96 - 0.99) |
|  | 2006 | 0.96 (0.95 - 0.97) | 0.95 (0.92 - 0.97) | 1.03 (0.97 - 1.09) | 0.98 (0.95 - 1.01) | 0.95 (0.93 - 0.96) |
|  | 2007 | 0.95 (0.93 - 0.97) | 0.97 (0.92 - 1.01) | 0.87 (0.79 - 0.96) | 0.95 (0.92 - 0.99) | 0.94 (0.91 - 0.97) |
|  | 2008 | 0.92 (0.90 - 0.94) | 0.94 (0.90 - 0.99) | 0.86 (0.78 - 0.95) | 0.93 (0.89 - 0.97) | 0.91 (0.88 - 0.94) |
|  | 2009 | 0.89 (0.87 - 0.92) | 0.91 (0.86 - 0.95) | 0.84 (0.76 - 0.93) | 0.91 (0.86 - 0.95) | 0.88 (0.85 - 0.92) |
|  | 2010 | 0.87 (0.84 - 0.89) | 0.87 (0.82 - 0.91) | 0.81 (0.73 - 0.91) | 0.89 (0.85 - 0.93) | 0.85 (0.82 - 0.88) |
|  | 2011 | 0.85 (0.82 - 0.87) | 0.84 (0.79 - 0.88) | 0.81 (0.73 - 0.91) | 0.88 (0.83 - 0.92) | 0.83 (0.80 - 0.86) |
|  | 2012 | 0.84 (0.81 - 0.86) | 0.81 (0.77 - 0.85) | 0.79 (0.71 - 0.89) | 0.86 (0.81 - 0.91) | 0.81 (0.78 - 0.84) |
|  | 2013 | 0.81 (0.79 - 0.84) | 0.78 (0.74 - 0.83) | 0.78 (0.69 - 0.88) | 0.84 (0.79 - 0.88) | 0.79 (0.76 - 0.82) |
|  | 2014 | 0.79 (0.77 - 0.82) | 0.76 (0.72 - 0.81) | 0.78 (0.69 - 0.88) | 0.82 (0.77 - 0.87) | 0.77 (0.74 - 0.81) |
|  | 2015 | 0.79 (0.76 - 0.81) | 0.75 (0.71 - 0.79) | 0.76 (0.67 - 0.86) | 0.80 (0.76 - 0.85) | 0.76 (0.73 - 0.80) |
|  | 2016 | 0.78 (0.76 - 0.81) | 0.74 (0.70 - 0.79) | 0.75 (0.66 - 0.85) | 0.79 (0.74 - 0.84) | 0.76 (0.72 - 0.79) |
|  | 2017 | 0.78 (0.75 - 0.80) | 0.73 (0.69 - 0.78) | 0.77 (0.68 - 0.88) | 0.78 (0.73 - 0.83) | 0.75 (0.72 - 0.78) |
|  | 2018 | 0.76 (0.74 - 0.79) | 0.71 (0.67 - 0.76) | 0.78 (0.68 - 0.89) | 0.76 (0.71 - 0.81) | 0.74 (0.71 - 0.77) |

Model was adjusted for number of inhabitants per neighbourhood.

Supplementary Table 5. Incidence rate ratio and 95% confidence interval as derived from negative binomial generalized estimating equations. Coefficients represent the average change in the neighbourhood counts of restaurants in the Netherlands. Coefficients are presented for the totality of neighbourhoods, as well as according to neighbourhood socio-economic status (SES) and urbanisation levels. (GEE) analysis.

|  |  | All neighbourhoods | Lowest SES neighbourhoods | Highest SES neighbourhoods | Lowest urbanisation | Highest urbanisation |
| --- | --- | --- | --- | --- | --- | --- |
| Food retailer category | Year | Coefficient | Coefficient | Coefficient | Coefficient | Coefficient |
|  |  | (95% CI) | (95% CI) | (95% CI) | (95% CI) | (95% CI) |
| Restaurants | 2004 | Reference | Reference | Reference | Reference | Reference |
|  | 2005 | 1.06 (1.04 - 1.07) | 1.03 (1.00 - 1.06) | 1.08 (1.04 - 1.12) | 1.08 (1.05 - 1.10) | 1.04 (1.03 - 1.05) |
|  | 2006 | 1.10 (1.08 - 1.11) | 1.03 (1.00 - 1.06) | 1.13 (1.09 - 1.17) | 1.16 (1.13 - 1.19) | 1.06 (1.04 - 1.08) |
|  | 2007 | 1.16 (1.13 - 1.19) | 1.12 (1.06 - 1.18) | 1.12 (1.06 - 1.19) | 1.21 (1.17 - 1.26) | 1.13 (1.10 - 1.18) |
|  | 2008 | 1.20 (1.17 - 1.23) | 1.16 (1.10 - 1.23) | 1.14 (1.08 - 1.22) | 1.25 (1.20 - 1.29) | 1.17 (1.13 - 1.22) |
|  | 2009 | 1.23 (1.19 - 1.26) | 1.21 (1.14 - 1.28) | 1.17 (1.09 - 1.25) | 1.28 (1.23 - 1.33) | 1.20 (1.16 - 1.25) |
|  | 2010 | 1.23 (1.20 - 1.26) | 1.20 (1.13 - 1.28) | 1.16 (1.09 - 1.24) | 1.29 (1.24 - 1.34) | 1.20 (1.16 - 1.25) |
|  | 2011 | 1.24 (1.20 - 1.27) | 1.23 (1.16 - 1.31) | 1.17 (1.09 - 1.25) | 1.28 (1.23 - 1.33) | 1.22 (1.18 - 1.27) |
|  | 2012 | 1.25 (1.21 - 1.28) | 1.23 (1.16 - 1.31) | 1.17 (1.10 - .25) | 1.29 (1.24 - 1.35) | 1.23 (1.18 - 1.28) |
|  | 2013 | 1.27 (1.23 - 1.30) | 1.25 (1.17 - 1.33) | 1.18 (1.11 - 1.27) | 1.33 (1.27 - 1.39) | 1.25 (1.20 - 1.30) |
|  | 2014 | 1.28 (1.24 - 1.32) | 1.23 (1.16 - 1.31) | 1.20 (1.12 - 1.29) | 1.34 (1.28 - 1.40) | 1.26 (1.21 - 1.32) |
|  | 2015 | 1.30 (1.26 - 1.34) | 1.25 (1.17 - 1.33) | 1.21 (1.13 - 1.30) | 1.36 (1.30 - 1.42) | 1.27 (1.22 - 1.33) |
|  | 2016 | 1.32 (1.28 - 1.37) | 1.28 (1.20 - 1.37) | 1.27 (1.19 - 1.37) | 1.38 (1.32 - 1.45) | 1.29 (1.23 - 1.36) |
|  | 2017 | 1.33 (1.29 - 1.38) | 1.30 (1.21 - 1.39) | 1.29 (1.20 - 1.40) | 1.40 (1.33 - 1.46) | 1.31 (1.24 - 1.38) |
|  | 2018 | 1.35 (1.30 - 1.40) | 1.32 (1.23 - 1.41) | 1.31 (1.21 - 1.41) | 1.41 (1.34 - 1.48) | 1.33 (1.26 - 1.40) |

Model was adjusted for number of inhabitants per neighbourhood.

Supplementary Table 6. Incidence rate ratio and 95% confidence interval as derived from negative binomial generalized estimating equations (GEE) analysis. Coefficients represent the average change in the neighbourhood counts of convenience stores in the Netherlands. Coefficients are presented for the totality of neighbourhoods, as well as according to neighbourhood socio-economic status (SES) and urbanisation levels.

|  |  | All neighbourhoods | Lowest SES neighbourhoods | Highest SES neighbourhoods | Lowest urbanisation | Highest urbanisation |
| --- | --- | --- | --- | --- | --- | --- |
| Food retailer category | Year | Coefficient | Coefficient | Coefficient | Coefficient | Coefficient |
|  |  | (95% CI) | (95% CI) | (95% CI) | (95% CI) | (95% CI) |
| Convenience stores | 2004 | Reference | Reference | Reference | Reference | Reference |
|  | 2005 | 1.08 (1.04 - 1.12) | 1.11 (1.05 - 1.19) | 1.06 (0.95 - 1.19) | 0.92 (0.86 - 0.98) | 1.15 (1.10 - 1.20) |
|  | 2006 | 1.10 (1.05 - 1.15) | 1.17 (1.09 - 1.26) | 1.00 (0.87 - 1.13) | 0.86 (0.79 - 0.94) | 1.22 (1.15 - 1.29) |
|  | 2007 | 1.11 (1.04 - 1.18) | 1.25 (1.14 - 1.38) | 0.91 (0.77 - 1.07) | 0.77 (0.70 - 0.86) | 1.29 (1.19 - 1.39) |
|  | 2008 | 1.09 (1.02 - 1.16) | 1.27 (1.14 - 1.41) | 0.81 (0.68 - 0.97) | 0.66 (0.58 - 0.74) | 1.31 (1.21 - 1.43) |
|  | 2009 | 1.05 (0.97 - 1.13) | 1.29 (1.16 - 1.45) | 0.73 (0.61 - 0.88) | 0.59 (0.51 - 0.67) | 1.30 (1.19 - 1.42) |
|  | 2010 | 1.04 (0.97 - 1.13) | 1.32 (1.17 - 1.49) | 0.68 (0.56 - 0.83) | 0.50 (0.43 - 0.59) | 1.34 (1.22 - 1.46) |
|  | 2011 | 1.02 (0.95 - 1.10) | 1.32 (1.17 - 1.48) | 0.60 (0.49 - 0.73) | 0.46 (0.39 - 0.54) | 1.34 (1.22 - 1.46) |
|  | 2012 | 1.13 (1.04 - 1.22) | 1.47 (1.30 - 1.65) | 0.60 (0.48 - 0.75) | 0.44 (0.38 - 0.52) | 1.50 (1.36 - 1.64) |
|  | 2013 | 1.12 (1.03 - 1.21) | 1.52 (1.35 - 1.72) | 0.57 (0.45 - 0.71) | 0.44 (0.37 - 0.52) | 1.48 (1.35 - 1.63) |
|  | 2014 | 1.13 (1.04 - 1.22) | 1.55 (1.38 - 1.75) | 0.56 (0.44 - 0.70) | 0.43 (0.36 - 0.51) | 1.50 (1.37 - 1.65) |
|  | 2015 | 1.11 (1.02 - 1.20) | 1.53 (1.35 - 1.73) | 0.49 (0.38 - 0.62) | 0.40 (0.33 - 0.48) | 1.47 (1.34 - 1.61) |
|  | 2016 | 1.14 (1.05 - 1.24) | 1.55 (1.36 - 1.76) | 0.54 (0.43 - 0.68) | 0.36 (0.30 - 0.45) | 1.53 (1.39 - 1.68) |
|  | 2017 | 1.14 (1.05 - 1.24) | 1.56 (1.37 - 1.78) | 0.55 (0.43 - 0.69) | 0.36 (0.29 - 0.45) | 1.51 (1.37 - 1.67) |
|  | 2018 | 1.13 (1.04 - 1.23) | 1.58 (1.38 - 1.80) | 0.54 (0.43 - 0.68) | 0.36 (0.29 - 0.45) | 1.50 (1.35 - 1.66) |

Model was adjusted for number of inhabitants per neighbourhood.
